# Macrophage colony-stimulating factor increases hepatic macrophage content, liver growth, and lipid accumulation in neonatal rats

**DOI:** 10.1152/ajpgi.00343.2017

**Published:** 2017-12-21

**Authors:** Clare Pridans, Kristin A. Sauter, Katharine M. Irvine, Gemma M. Davis, Lucas Lefevre, Anna Raper, Rocio Rojo, Ajit J. Nirmal, Philippa Beard, Michael Cheeseman, David A. Hume

**Affiliations:** ^1^The Roslin Institute, University of Edinburgh, Edinburgh, United Kingdom; ^2^Medical Research Council Centre for Inflammation Research, University of Edinburgh, The Queen's Medical Research Institute, Edinburgh, United Kingdom; ^3^Mater Research-University of Queensland, Translational Research Institute, Woolloongabba, Australia; ^4^The Pirbright Institute, Surrey, United Kingdom

**Keywords:** CSF1, CSF1R, Kupffer cells, M-CSF, myeloid

## Abstract

Signaling via the colony-stimulating factor 1 receptor (CSF1R) controls the survival, differentiation, and proliferation of macrophages. Mutations in CSF1 or CSF1R in mice and rats have pleiotropic effects on postnatal somatic growth. We tested the possible application of pig CSF1-Fc fusion protein as a therapy for low birth weight (LBW) at term, using a model based on maternal dexamethasone treatment in rats. Neonatal CSF1-Fc treatment did not alter somatic growth and did not increase the blood monocyte count. Instead, there was a substantial increase in the size of liver in both control and LBW rats, and the treatment greatly exacerbated lipid droplet accumulation seen in the dexamethasone LBW model. These effects were reversed upon cessation of treatment. Transcriptional profiling of the livers supported histochemical evidence of a large increase in macrophages with a resident Kupffer cell phenotype and revealed increased expression of many genes implicated in lipid droplet formation. There was no further increase in hepatocyte proliferation over the already high rates in neonatal liver. In conclusion, treatment of neonatal rats with CSF1-Fc caused an increase in liver size and hepatic lipid accumulation, due to Kupffer cell expansion and/or activation rather than hepatocyte proliferation. Increased liver macrophage numbers and expression of endocytic receptors could mitigate defective clearance functions in neonates.

**NEW & NOTEWORTHY** This study is based on extensive studies in mice and pigs of the role of CSF1/CSF1R in macrophage development and postnatal growth. We extended the study to neonatal rats as a possible therapy for low birth weight. Unlike our previous studies in mice and pigs, there was no increase in hepatocyte proliferation and no increase in monocyte numbers. Instead, neonatal rats treated with CSF1 displayed reversible hepatic steatosis and Kupffer cell expansion.

## INTRODUCTION

Signals initiated following binding of macrophage colony-stimulating factor 1 (CSF1) or IL-34 to a shared receptor (CSF1R) control the survival, differentiation, and proliferation of cells of the mononuclear phagocyte lineage (6, 21, 24). Mutation of CSF1 in rats or mice produces a global deficiency of macrophage numbers in most tissues, whereas IL-34 appears to be required more specifically for macrophages of the brain (microglia) and skin (Langerhans cells) (55). Mutation of the receptor CSF1R, which ablates the response to both ligands, has a more penetrant phenotype in mice (8) and rats (C. Pridans, A. Raper, G. M. Davis, J. Alves, K. A. Sauter, L. Lefevre, T. Regan, S. Meek, L. Sutherland, A. J. Thomson, S. Clohisey, R. Rojo, Z. M. Lisowski, R. Wallace, K. Grabert, K. R. Upton, Y. T. Tsai, D. Brown, L. B. Smith, K. M. Summers, N. A. Mabbott, P. Piccardo, M. T. Cheeseman, T. Burdon, D. A. Hume, unpublished observations). The requirement for continuous CSF1R signaling is retained in adult mice, in that treatment with an anti-CSF1R antibody depletes tissue macrophages from the majority of organs (26). However, the availability of CSF1R ligands in vivo is not saturating. Administration of recombinant human CSF1 to mice expanded the circulating blood monocyte and tissue macrophage populations (22). These studies subsequently led to confirmation of biological efficacy in human patients (reviewed in Ref. 21). The circulating CSF1 concentration is controlled by CSF1R-mediated endocytic clearance by macrophages of the liver and spleen (3), providing a homeostatic loop in which tissue macrophages control monocyte production from the bone marrow (BM) (24). Accordingly, anti-CSF1R treatment or mutation of the receptor increases circulating CSF1 concentration (8, 26). Macrophages throughout the body occupy defined niches or territories (17), and the local availability of CSF1 in tissues may be one determinant of the size/boundary of those territories and local self-renewal of macrophages (24).

Mutation of CSF1 or CSF1R in mice or rats produces a severe postnatal growth retardation due, at least in part, to diminished production of the somatic growth factor insulin-like growth factor 1 (IGF-1). Hence, CSF1/CSF1R could be considered part of the growth hormone/IGF-1 axis (15). Consistent with that hypothesis, treatment of newborn mice with recombinant CSF1 produced an increase in somatic growth rate 30 days after birth, associated with increased *Igf1* expression (1). To increase the circulating half-life, and potential efficacy of CSF1, we produced a pig CSF1-Fc fusion protein (14). Pig CSF1 is equally active in humans and mice (13), and the pig provides a preclinical model in which to evaluate therapeutic potential (9).

CSF1-Fc treatment of adult mice produced a rapid increase in the size of the liver associated with extensive hepatocyte proliferation (14). The *Csf1r* gene is expressed solely in cells of the macrophage lineage, and the transcriptional regulation has been studied in detail (39). A *Csf1r*-EGFP marker provides a marker for Kupffer cells in the liver (44), and treatment of mice with a neutralizing anti-CSF1R antibody completely depletes the transgene-positive Kupffer cell population (26). By contrast, hepatic parenchymal cells in vivo, or in vitro, do not possess any detectable CSF1 binding activity (3). Hence, the effects of CSF1-Fc on the liver must be mediated indirectly through interactions between macrophages and other liver cells. Subsequent studies confirmed that the liver controls circulating CSF1 concentration in humans, and CSF1-Fc treatment can promote liver regeneration in mouse models (50). CSF1-Fc treatment also promoted liver growth when administered to pigs (46). On the basis of these findings, CSF1 was proposed to be a significant component of the so-called hepatostat (46), which controls the homeostatic size of the liver (30). Treatment of newborn piglets with CSF1-Fc did not produce the increase in body weight gain that had previously been observed in mice (46). Pigs are a precocial species, and commercial animals have been genetically selected for very rapid growth rate. We therefore decided to investigate the impacts of CSF1-Fc in rats.

Our initial experiments suggested that CSF1-Fc treatment did not increase postnatal growth in rats, but we did not determine whether this was because the CSF1 was inactive or because it was already saturating. We considered the possibility that CSF1-Fc might be more efficient in animals with low birth weight (LBW). To this end, we utilized a well-studied model of maternal stress in which pregnant rats are injected with glucocorticoids in the third week of pregnancy (10, 11). This treatment produces lifelong impacts on several organ systems, notably predisposing to lipid accumulation in the liver (20, 53). Here, we show that pig CSF1-Fc is active in neonatal rats, and produces a significant expansion of mononuclear phagocytes in the liver and spleen, associated with hepatosplenomegaly. However, the treatment also caused an unexpected, large, but reversible increase in fat deposition in the liver.

## EXPERIMENTAL PROCEDURES

### 

#### Rats.

Experiments were carried out under the authority of a UK Home Office Project Licence under the regulations of the Animals (Scientific Procedures) Act 1986, approved by The Roslin Institute and The University of Edinburgh Animal Welfare and Ethical Review Body. Rats were housed in Techniplast GM1500 Green Line individually ventilated cages lined with Eco pure aspen chip (2HK) and fed T.2914.12 irradiated 14% protein (Envigo, UK). Enrichment was provided with aspen chew sticks and sizzle pet (LBS Biotechnology, UK). Sprague-Dawley rats were injected subcutaneously with 0.15 mg/kg dexamethasone (Dexadreson) or PBS on *days 14–21* of pregnancy. At parturition, pups were given to foster dams and injected subcutaneously with either 1 µg/g porcine CSF1-Fc (14) or PBS for 5 days. Injections occurred during the light cycle. Pups were weighed daily, and blood was collected by cardiac puncture following euthanasia at *day 6*. A separate cohort was treated as above and euthanized on *day 32* (recovery experiment).

#### Bone marrow macrophage viability assay.

Bone marrow (BM) was isolated from adult male Sprague-Dawley rats. Cells were plated at 3 × 10^5^ cells/well of a 96-well plate in complete medium either without growth factors, with 10^4^ U/ml (100 ng/ml) recombinant human CSF1, or various concentrations of porcine CSF1-Fc (pCSF1-Fc) and incubated at 37°C, 5% CO_2_ for 7 days for macrophage differentiation. MTT was added directly to growth medium at a final concentration of 0.5 mg/ml, and the plate was incubated at 37°C for 3 h. Solubilization of tetrazolium salt was achieved with a solution of 10% SDS-50% isopropanol-0.01 M HCl at 37°C overnight. The plates were read at 570 nm.

#### Histology and immunohistochemistry.

Spleens and livers were weighed and then fixed in 10% buffered formalin and processed into paraffin by the Histopathology Department at the Royal (Dick) School of Veterinary Studies using standard procedures. Slides were stained with hematoxylin and eosin (H&E). Immunohistochemistry (IHC) was performed with mouse anti-rat CD68 (Clone ED1, 1:500, Bio-Rad). For Oil red O staining, formalin-fixed livers were placed in 18% sucrose at 4°C overnight and cryosections prepared as described in (45). Staining was performed as described in Ref. 29. Sections were imaged using a Nanozoomer digital scanner and viewed using NDP.view 2 (Hamamatsu, Japan). Oil red O staining was imaged with standard light microscopy using ZEN software (Zeiss).

#### Image analysis.

Image analysis was performed in ImageJ using six images per organ. CD68 staining was quantified in spleen and liver based on the threshold (1–150). The size and abundance of lipid particles in the liver were quantified using H&E-stained sections by particle analysis (5 µm–infinity and 0.45–1 circularity).

#### Blood analysis.

All tests were performed by the Clinical Pathology department at the Royal (Dick) School of Veterinary Studies. Blood was collected into 0.5 ml EDTA tubes (Teklab). Total WBC, RBC, monocytes, lymphocytes and neutrophils were measured on the ABX Pentra 60 hematology analyzer (Hariba Medical). EDTA-plasma was used for a Total Bile Acids detection kit (Diazyme) and measured on an ILab 650 Biochemistry Analyser.

#### Statistical analysis.

Data were analyzed using a Mann-Whitney test. Results are presented as box and whisker plots (horizontal line within the box indicates the median, boundaries of the box indicate 25th and 75th %ile, and whiskers indicate highest and lowest values of the results).Weights (*days 0–6*) were analyzed using repeated-measures two-way ANOVA. All analyses were performed using GraphPad Prism 5.0 (GraphPad Software). A *P* value < 0.05 was considered statistically significant.

#### Microarray.

RNA was extracted from rat liver using TRIzol (ThermoFisher Scientific) followed by purification using the RNeasy Mini Kit (Qiagen) according to the manufacturer’s protocols. RNA integrity and quality were assessed using the RNA ScreenTape Kit on the Agilent 2200 TapeStation. Samples with a RNA integrity number greater than 7 were used for microarray. Microarray was performed by Edinburgh Genomics (Edinburgh, UK) using Affymetrix Rat Gene 2.1 ST Array plates, and Expression Console 1.4.1.46 was used for quality control following amplification.

The signal intensities of microarray results were summarized to probe sets and normalized using robust multi-array average (RMA) in R, using the Affymetrix Transcription Analysis Console. The RMA-normalized data were loaded into the network analysis tool Miru (Kajeka, UK) for further analysis alongside that of BM-derived macrophage (BMDM) data generated from Dark Agouti rats (our unpublished data). A Pearson correlation matrix of a gene-to-gene profile comparison was used to filter for expression correlation relationships of ≥0.96 across the microarray samples. Nodes within the network graph represent transcripts and the edges between them represent expression correlations above the set threshold. To identify groups of tightly co-expressed genes, the graph was clustered using the graph-based clustering algorithm MCL set at an inflation value (which determines the granularity of the clusters) of 1.8. Gene lists associated with the clusters were exported for GO annotation analysis (Biological and Metabolic Processes Level-FAT) using DAVID (Database for Annotation, Visualization, and Integrated Discovery). GEO accession no. GSE104584. CIBERSORT analysis (http://cibersort.stanford.edu/ (32),) was performed using the LM22 expression matrix, which contains expression signatures for 22 human hematopoietic cell types/states and default settings. The macrophage-lineage expression profiles in the LM22 matrix are derived from freshly isolated monocytes from peripheral blood mononuclear cells, unpolarized macrophages (M0, generated by differentiation of monocytes in human serum for 7 days), classically activated macrophages (M1, generated by differentiation of monocytes in CSF1 for 7 days followed by stimulation with LPS and IFNγ for 18 h), and alternatively activated macrophages (M2, generated by differentiation of monocytes in CSF1 for 7 days followed by stimulation with IL-4 for 18 h) (32). Deconvolution of liver samples was associated with global *P* values of 0.0 (for CSF1-Fc-treated samples) and 0.07–0.09 (for PBS-treated samples), indicating strong goodness of fit for the deconvolution.

## RESULTS

### 

#### Pig CSF1-Fc is active on rat macrophages but does not affect postnatal growth.

**Fig. 1.**
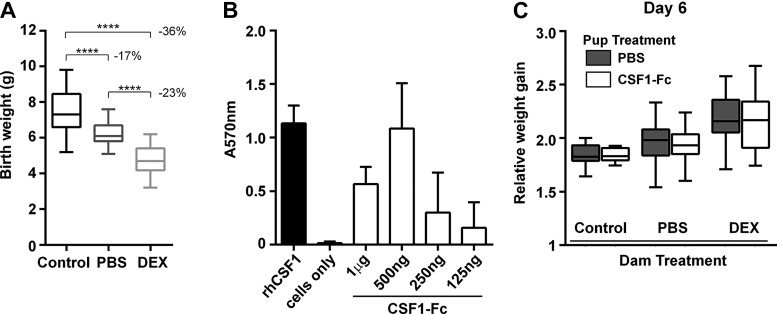
(CSF1-Fc) does not promote weight gain in neonatal rats. *A*: rats were injected with PBS or dexamethasone (DEX) on *days 14–21* of pregnancy. Control dams were not injected. Box and whisker plots of birth weight; *n* = 37–65. Results were analyzed with a Mann-Whitney test. ****P < 0.0001. *B*: bone marrow (BM) from male rats was cultured in 100 ng/ml recombinant human CSF1 (rhCSF1), porcine CSF1-Fc, or without growth factor (cells only) for 7 days. MTT was used to assess cell viability; *n* = 2 repeat experiments. Graphs show means +SE. *C*: offspring were injected with PBS or 1 µg/g CSF1 on *days 0–5* and weighed daily. Box and whisker plots of weight at *day 6*; *n* = 7–19. Results were analyzed by repeated-measures two-way ANOVA (*days 0–6*).

Prenatal dexamethasone (DEX) treatment of rats produces 8.5–25% lower birth weight in different studies (7, 33) compared with vehicle-injected rats. To establish the model, Sprague-Dawley rats were injected on *days 14–21* of pregnancy with either DEX or PBS. Injection of PBS was sufficient to reduce the average birth weight. DEX treatment lowered birth rate further, producing a 36% reduction relative to noninjected controls (Fig. 1*A*). The activity of pig CSF1-Fc on rat BM was comparable to that of the human recombinant protein (Fig. 1*B*). Newborn pups were injected with either PBS or 1 mg/kg pCSF1-Fc subcutaneously from birth to postnatal *day 5*, weighed daily, and analyzed on *day 6*. The smaller pups from PBS and DEX-treated dams showed a somewhat greater weight gain, apparently recovering their initial weight disadvantage. However, pCSF1-Fc treatment did not increase or decrease weight gain in any of the treatment groups (Fig. 1*C*).

#### Impact of CSF1-Fc treatment on circulating hematopoietic cells in neonatal rats.

**Fig. 2.**
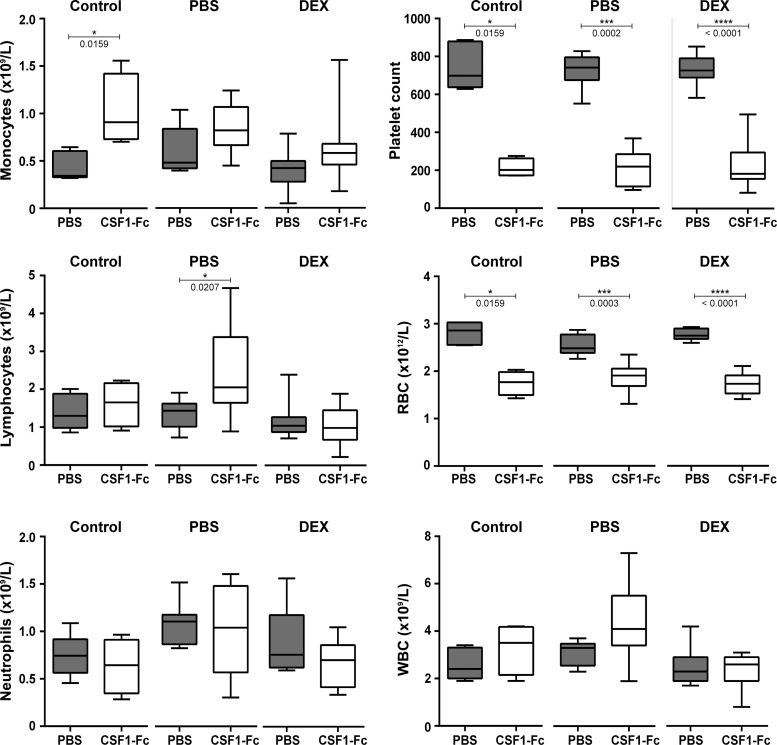
Effect of CSF1-Fc treatment on the blood of neonatal rats. Rats were injected with PBS or DEX on *days 14–21* of pregnancy. Control dams were not injected. Offspring were treated with PBS or 1 µg/g porcine CSF1-Fc on *days 0–5*. EDTA-blood was obtained from rats at *day 6*; *n* = 4–5 (control), *8* (PBS), and *11–15* (DEX). Results were analyzed with a Mann-Whitney test. *P* values are indicated on the box and whisker plots.

In mice and humans, there is a postnatal surge in serum CSF1 (40, 41). We considered the possibility that CSF1 levels in all rat pups might already be saturating and/or that CSF1-Fc was not active in neonatal rats. Treatment of adult mice or weaner pigs with pCSF1-Fc greatly increased the number of circulating monocytes (14, 46), but we had not previously examined this in neonates. The blood monocyte numbers were marginally affected by the treatment (Fig. 2). Changes in total blood leukocytes, lymphocytes, and neutrophils were also marginal (Fig. 2). However, CSF1-Fc was clearly active. In initial studies of infusion of CSF1 in human patients, the dose-limiting toxicity was thrombocytopenia (23), and we also observed reduced platelet numbers in mice and pigs treated with CSF1-Fc. In the CSF1-Fc-treated neonatal rats there was a 70% reduced platelet count and a 20–30% reduction in red blood cells in all groups (Fig. 2).

#### CSF1-Fc causes hepatosplenomegaly in neonatal rats.

**Fig. 3.**
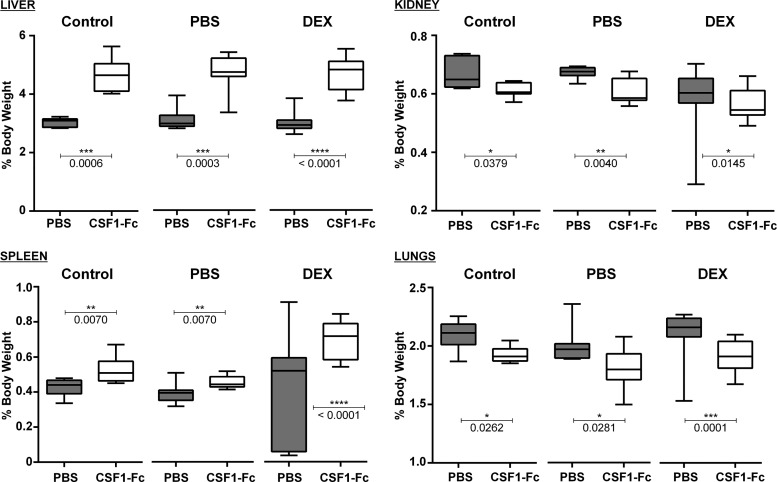
Effect of CSF1-Fc treatment on organ weights of neonatal rats. Rats were injected with PBS or DEX on *days 14–21* of pregnancy. Control dams were not injected. Offspring were treated with PBS or 1 µg/g porcine CSF1-Fc on *days 0–5*. Organs were weighed at *day 6*; *n* = 7 (control), *8* (PBS), and *15–17* (DEX). Results were analyzed with a Mann-Whitney test. *P* values are indicated on the box and whisker plots.

Treatment of adult mice with CSF1-Fc caused hepatosplenomegaly, associated with the proliferation of hepatocytes in the liver (14). Although the size of major organs did not differ between normal and LBW rats, CSF1-Fc caused a >30% increase in the relative size of the liver in each treated group (Fig. 3). In adult mice, there is very little hepatocyte proliferation at steady state, and CSF1-Fc treatment caused a massive increase in staining with Ki67 or proliferating cell nuclear antigen (14). In weaner pigs, the baseline proliferation of hepatocytes was higher, but an increase in response to CSF1-Fc was still evident. In rat pups, there was extensive staining for proliferating cell nuclear antigen, even in untreated animals, and no additional effect of CSF1-Fc was detected (not shown).

#### CSF1-Fc treatment increases lipid accumulation in neonatal rats.

**Fig. 4.**
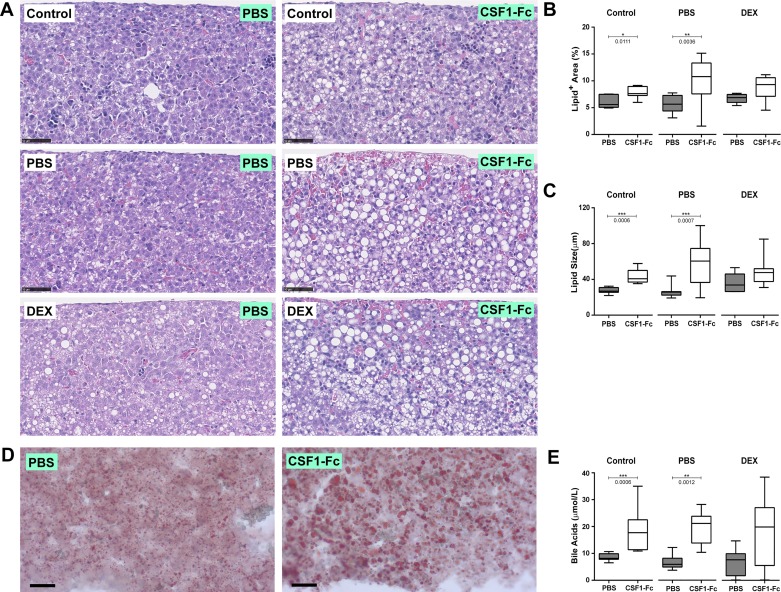
CSF1-Fc treatment causes lipid accumulation in livers of neonatal rats. Rats were injected with PBS or DEX on *days 14–21* of pregnancy. Control dams were not injected. Offspring were treated with PBS or 1 µg/g porcine CSF1-Fc on *days 0–5*. *A*: representative hematoxylin and Eosin (H&E) sections of formalin-fixed paraffin-embedded livers. ImageJ was used to quantify the percentage area of lipids (*B*) and lipid size (*C*), using 6 H&E images per liver; *n* = 7 (control), 11–12 (PBS), and 7 (DEX). *D*: representative Oil red O staining of frozen liver sections. *E*: EDTA-plasma was tested for bile acids; *n* = 7–8 for all groups. All results were analyzed with a Mann-Whitney test. *P* values are indicated on the box and whisker plots.

Examination of liver histology revealed the likely reason for the increased size of the liver in response to CSF1-Fc treatment, as a large accumulation of lipid droplets was evident in each group (Fig. 4*A*). Consistent with published data (42), lipid accumulation was evident in untreated pups from DEX-treated dams. The abundance and apparent size of these droplets were further exacerbated by CSF1-Fc treatment (Fig. 4, *A–C*). The impact of CSF1-Fc treatment was confirmed by Oil-red O staining (Fig. 4*D*). Lipid accumulation in the liver is commonly accompanied by accumulation of bile acids, which have themselves been attributed roles in fetal liver hematopoiesis (49). In all of the treated groups there was a two- to threefold increase in the circulating bile acids in response to CSF1-Fc treatment (Fig. 4*E*).

#### CSF1-Fc increases hepatic and splenic macrophage numbers.

**Fig. 5.**
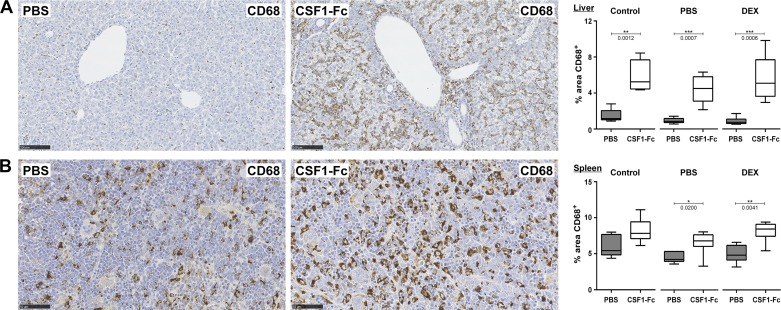
Treatment of neonatal rats with CSF1-Fc causes an increase in liver and splenic macrophages. Rats were injected with PBS or DEX on *days 14–21* of pregnancy. Control dams were not injected. Offspring were treated with PBS or 1 µg/g porcine CSF1-Fc on *days 0–5*. Organs were collected at *day 6*. Representative images of formalin-fixed, paraffin-embedded livers (*A*) and spleens (*B*) stained with an antibody against CD68. Sections shown are from neonatal rats born to PBS-injected dams. Percentage area of CD68^+^ staining was calculated with ImageJ using 6 images per organ; *n* = 6–8 for all groups. All results were analyzed with a Mann-Whitney test. *P* values are indicated on the box and whisker plots.

In light of the relative lack of impact of CSF1-Fc on blood monocyte counts, we investigated whether the treatment increased tissue macrophage numbers in the liver and spleen. As shown in Fig. 5, there was a three- to fourfold increase in CD68^+^-positive cells in the liver and a smaller increase, from a higher basal level, in the spleen. LBW following maternal DEX treatment did not affect macrophage numbers in either organ or the response to CSF1-Fc.

#### CSF1-Fc-regulated gene expression in neonatal rat liver.

**Fig. 6.**
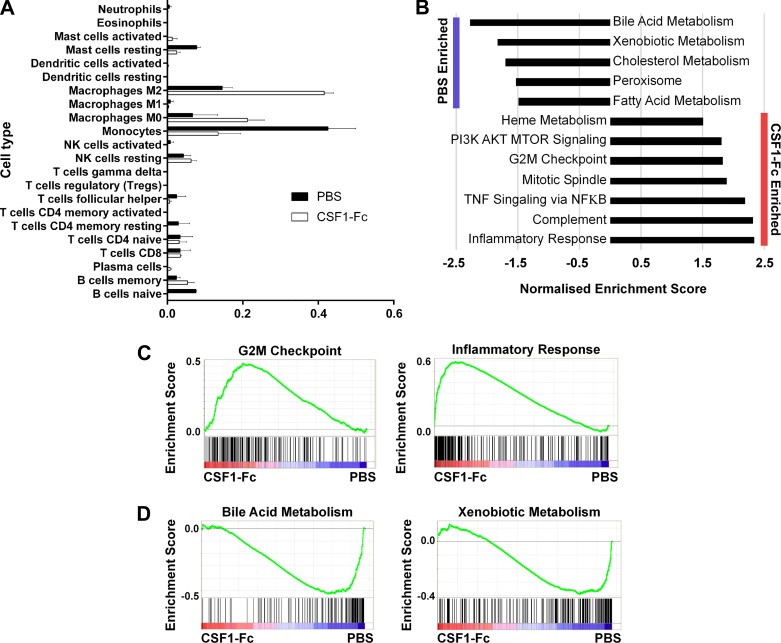
Gene set enrichment analysis of neonatal rat livers. Rats were injected with PBS on *days 14–21* of pregnancy, and offspring were treated with PBS or 1 µg/g porcine CSF1-Fc on *days 0–5*. Livers were collected at *day 6* for RNA isolation and subsequent microarray analysis. *A*: relative proportion of hematopoietic cell types in PBS- and CSF1-Fc-treated livers predicted using CIBERSORT. *B*: significantly enriched hallmark gene sets were determined by Gene Set Enrichment Analysis (FDR *q*-value <0.05). Example gene set enrichment plots for CSF1-Fc- (C) and PBS- (*D*) treated neonatal rat livers. Expression data were ranked according to normalized expression in CSF1-Fc vs. PBS-treated liver (indicated by red-blue bars), and the gene set of interest was mapped onto this profile (filled bars) to determine enrichment scores (green lines).

In adult mice and weaner pigs, the mechanism(s) underlying the impact of CSF1-Fc treatment on liver growth was dissected by array profiling intact liver in parallel with BMDM (14, 46). Analysis of the data as a network graph, using Biolayout Express^3D^ (now Graphia^PRO^), enabled the identification of clusters of genes that were induced by CSF1, including those that are shared with BMDM and those induced specifically in the liver. The latter set would include genes that are expressed uniquely by the macrophages of the liver (28). We generated mRNA expression array profiles for rat BMDM and for the livers from control (PBS-) and CSF1-Fc-treated control rats (born to PBS-treated dams). The complete primary data set is provided in Supplementary Table S1 (supplementary tables online only), with average expression of each transcript and fold change between PBS- and CSF1-Fc-treated rat livers. The set of ~650 transcripts increased at least twofold in the liver by CSF1-Fc treatment (Supplementary Table S1) is consistent with the three- to fourfold increase in macrophage content shown in Fig. 5. It includes markers such as *Adgre1* (F4/80), *Fcgr1a* (Cd64), and *Cd14* and multiple transcription factor genes (*Fli1, Nr1h3, Tfec, Elf4, Irf8, Spi1, SpiC, Runx1, Runx3, Klf4, Etv1, Etv5, Irf5, Fos*), implicated in regulated expression of *Csf1r* and/or macrophage differentiation (39). None of these transcription factors was enriched in the liver relative to BMDM. *Id3*, which was implicated in Kupffer cell differentiation in mice (28), was constitutively expressed in neonatal rat liver and not elevated further by CSF1-Fc, suggesting that it is not specifically associated with the macrophage population. To gain insight into the broad impact of CSF1-Fc treatment on liver macrophages, and other hematopoietic cell populations, we used CIBERSORT, a tool for characterizing cellular composition of complex tissues from gene expression profiles (32). Livers from control mice were predicted to contain a higher proportion of monocyte-like cells compared with treated mice, which might reflect liver macrophage immaturity in neonatal rats (Fig. 6*A*). CSF1-Fc treatment led to a significant reduction in the monocyte expression signature, with a concomitant increase in mature macrophage (M0) and alternatively activated macrophage (M2) signatures (Fig. 6*A*). No significant changes in other liver hematopoietic cell populations were observed, confirming the specific impact of CSF1-Fc on the macrophage lineage.

**Fig. 7.**
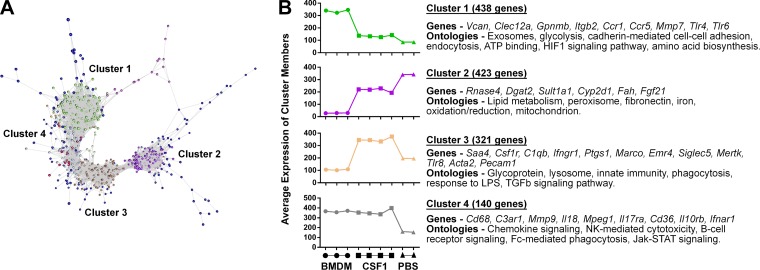
CSF1-Fc-regulated gene expression in neonatal rat liver. Rats were injected with PBS on *days 14–21* of pregnancy, and offspring were treated with PBS or 1 µg/g porcine CSF1-Fc on *days 0–5*. Livers were collected at *day 6* for RNA isolation and subsequent microarray analysis. *A*: normalized data were clustered with gene expression data from bone marrow-derived macrophages (BMDM) by Spearman correlation using Graphia^PRO^. *B*: graphs show average expression of clusters in BMDM and CSF1-Fc-, and PBS -treated neonatal rats born to PBS-injected dams.

Gene set enrichment analysis (GSEA) (Fig. 6, *B–D*) revealed relative enrichment of cell cyle and inflammation-related terms in the CSF1-Fc-treated livers and of lipid and xenobiotic metabolism in control livers. A network graph created with Graphia^PRO^ generated similar liver- and macrophage-associated clusters to conventional hierarchical clustering (Fig. 7, *A* and *B*). The average profiles of the four largest clusters and representative transcripts are shown. The complete transcript list for each of these clusters is provided in Supplementary Table S2. The implications of the set of inducible genes and each of the clusters and their relationship to the macrophage recruitment and accumulation of lipids are discussed below.

#### CSF1-Fc-induced macrophage expansion, hepatosplenomegaly, and lipid accumulation are reversible.

**Fig. 8.**
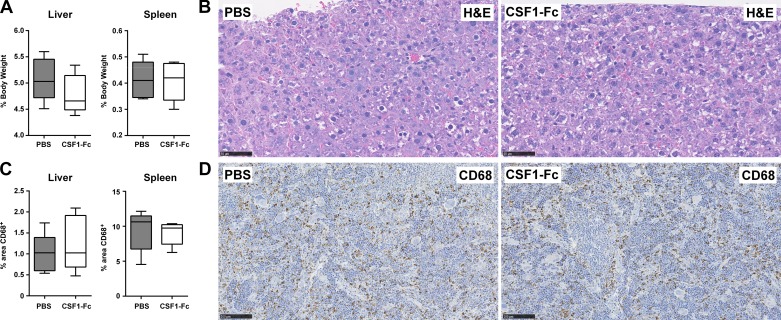
Cessation of CSF1-Fc treatment reverts organ size, lipid accumulation, and macrophage numbers to control levels. Rats were injected with DEX on *days 14–21* of pregnancy, and offspring were treated with PBS or 1 µg/g porcine CSF1-Fc on *days 0–5*. *A*: organs were collected and weighed 27 days following the last injection. *B*: representative H&E sections of formalin-fixed, paraffin-embedded livers. *C*: box and whisker plots showing percentage area of CD68^+^ staining as calculated by ImageJ using 6 images per organ; *n* = 5 for all groups. *D*: representative CD68 immunohistochemistry of formalin-fixed, paraffin-embedded spleens.

Having observed the impact of CSF1-Fc treatment on the growth of the liver and fat deposition, we asked whether the effect was reversible upon cessation of treatment. Control and treated animals were allowed to recover for an additional 27 days following treatment. As shown in Fig. 8, at that time there was no longer any difference between the groups in terms of relative liver and spleen size; the content of CD68^+^ cells in the spleen was normalized, and fat deposition in the liver was no longer evident.

## DISCUSSION

Concentrations of CSF1 were found to be higher in fetal than in maternal blood throughout mouse gestation and to peak around the time of birth, at which time there was also a peak of CSF1 protein in the liver (41). In humans, CSF1 is also elevated in the embryonic circulation relative to the maternal, and there was a two- to threefold increase in the first few days after a full-term birth (40). In the current study, we examined the possible roles of that increase in available CSF1 by administering an exogenous source for the first 5 days of life in rats.

The FANTOM5 consortium produced detailed time courses of the mRNA expression profile of developing mouse organs based on Cap Analysis of Gene Expression. Analysis of these data revealed a signature of increased macrophage content with time of development (51). The macrophages of the mouse liver displayed a unique gene expression profile including endocytic receptors (*Clec4f* and *Timd4*). Their expression in the liver escalated rapidly between neonatal *day 0* and *day 7*, consistent with a role for the postnatal surge in the liver and circulating CSF1 in the expansion of this population. Correlation-based network analysis of gene expression profiles of CSF1-Fc-treated and control livers from neonatal rats, alongside BMDM, revealed four clear clusters of liver- and macrophage-associated genes. *Cluster 3* (Fig. 7*B* and Supplementary Table S2) contained transcripts that were relatively low or absent in BMDM, higher in liver from control mice, and elevated four- to fivefold further in response to CSF1-Fc. The cluster included *Clec4f*, *Marco, Vsig4*, and *Timd4*, identified in mice as definitive markers of resident Kupffer cells, compared with monocytes and monocyte-derived macrophages (47, 54). The cluster also contained Kupffer cell-associated transcripts *Cd163*, ferroportin (*Slc40a1*), the heme transporter *Slc48a1*, and heme oxygenase (*Hmox1*), involved in the elimination of senescent red cells and recycling of heme iron (31). Two other macrophage-related clusters were identified in the data. *Cluster 4* (Fig. 7*B*) was expressed highly in BMDM, and strongly-inducible in the liver in response to CSF1-Fc. The cluster included *Cd68* (consistent with Fig. 5), *Adgre1* (F4/80), *Csf1r*, and *Mpeg1*. *Cluster 1* (Fig. 7*B*) contained macrophage-related genes (e.g., *Gpnmb*) that were strongly expressed in BMDM and induced to a lesser extent by CSF1-Fc treatment. *Cluster 2* contained transcripts that were not detected in BMDM, and were reduced in CSF1-Fc-treated livers. The reduction in their expression was not simply due to dilution by the macrophage-associated transcripts. Hepatocyte-specific genes such as *Alb* and *Afp* were unaffected (Supplementary Table S1).

In adult mice, CSF1-Fc treatment promoted the proliferation of both resident Kupffer cells and infiltrating monocytes (50). Monocyte recruitment depends on *Ccl2* signaling via *Ccr2*, but, unlike in mice, neither gene was induced by CSF1-Fc in rats. Monocyte markers such as *Spn* (*Cd43*) (19) and *Ly6C* were readily detected in the liver but were not increased further by CSF1-Fc (Supplementary Table S1). CIBERSORT analysis identified a reduced monocyte expression signature in CSF1-Fc-treated livers (Fig. 6*A*). Taken together with the large increase in expression of liver macrophage-specific genes such as *Timd4* and *Vsig4*, and the lack of a substantial increase in circulating monocyte numbers (Fig. 2), these findings suggest that most of the increase in liver macrophages arises from proliferation of resident Kupffer cells. Some of the regulated genes in Supplementary Table S1, such as *Cd163, Cd206*, and *Msr1 (Cd204*) have been regarded as markers of M2-macrophage polarization in rats (52) (12), and the inducible gene profile also includes members of the *Tgfb* family (*Tgfb1, Tgfb3*), which are M2-associated cytokines (57). However, *Cd163* is known to be expressed constitutively by Kupffer cells and many resident macrophages in rats (37). These genes are all expressed constitutively in the naïve liver, and they are not increased to any greater extent than the Kupffer cell markers or generic macrophage-associated transcripts such as *Cd14, Cd68, Adgre1*, and *Csf1r*. By contrast, IL-4 target genes such as *Tgm2, Arg1* and *Retnla* (27) were not increased in the treated liver, suggesting the “M2” expression signature detected in CSF1-Fc-treated liver by CIBERSORT deconvolution analysis relates to the Kupffer cell profile mentioned above rather than IL4-mediated alternative activation. Accordingly, CSF1-Fc treatment appears to act solely to promote expansion and maturation of the resident macrophage population of the neonatal liver, reflected by increased mature macrophage expression signatures (M0, M2).

CSF1-Fc treatment did not increase hepatocyte proliferation in neonatal rats. Hence, the increase in liver size was mainly due to the extensive lipid droplet formation. Genes such as *Pcna*, the transcription factor *FoxM1*, and cyclins (e.g., *Ccna2*), were expressed constitutively in both rat BMDM, which are actively proliferating, and in the liver, regardless of CSF1-Fc treatment (Supplementary Table S1). Nevertheless, the GSEA in Fig. 6 indicates enrichment for cell cycle-associated genes in response to CSF1-Fc, which is likely to be associated with proliferation of the macrophages (50). In adult mice, the proliferation of hepatocytes is dependent in part on inflammatory cytokines such as IL-6 and TNF produced by incoming Ccr2-dependent monocytes in response to exposure to portal blood (14, 50). The increased expression of classical LPS-responsive proinflammatory genes was detectable in expression profiles of liver from CSF1-Fc-treated mice and pigs (14, 50). By contrast, no induction of mRNA encoding these inflammatory cytokines was seen in the CSF1-Fc-treated neonatal rat livers. We suggest that the lack of inflammatory cytokine induction in neonatal liver reflects the absence of monocyte recruitment.

CSF1-Fc treatment alone was sufficient to promote lipid droplet accumulation, and the comparative gene expression profiles in Supplementary Table S1 may give clues as to the underlying mechanism. The biogenesis of lipid droplets has been reviewed by Pol et al. (36). Most genes involved [e.g., *Acscl1,3*, and *4*, *Aup1*, *Dgat2, Spg20, Cct1, Pemt*, perilipins (*Plin2, Plin3*), and *Bscl2* (aka *seipin*)] were expressed in neonatal rat liver but were not increased further by CSF1-Fc (Supplementary Table S1). One possible site of regulation is the uptake of fatty acids. However, the two most highly expressed hepatocyte fatty acid transporters, encoded by *Slc27a2* and *Slc27a5*, were each somewhat downregulated in CSF1-Fc treated livers, as were the major fatty acid binding proteins *Fabp1*, *Fabp5*, and *Fabp 7.* The fatty acid translocase *Cd36* was elevated four- to fivefold in the CSF1-Fc-treated livers. *Cd36* is highly expressed in macrophages; however it is also implicated in fatty acid uptake in hepatocytes in rats (5) and mice (56), suggesting that some of this induction may occur in hepatocytes. Hepatocyte-specific conditional deletion of *Cd36* in mice was shown recently to greatly attenuate lipid accumulation in two models of hepatic steatosis (56). The active cholesterol transporter *Abcg1* (34), less well-studied cholesterol-sensitive transporter *Abca9* (35), phospholipid transfer protein *Pltp* (2), and the insulin-converting enzyme *Pcsk1*, linked to regulated lipid droplet accumulation in adipocytes (43), were also upregulated in response to CSF1. Downstream of lipid uptake, acyl-CoA synthesis is an essential event in lipid droplet formation, in which the Acsl family are implicated (36). The long- and short-chain acyl-CoA synthetases *Acsbg1, Acsf2*, and *Acss1* and the remodeling enzyme *Lpcat2* (18) were each strongly increased by CSF1-Fc treatment. Ceramide kinase (*Cerk*), an upstream regulator of phospholipase A_2_ activation and lipid droplet formation (16), was also increased more than twofold by CSF1-Fc. The set of genes downregulated by CSF1-Fc (*cluster 2*) may also contribute to lipid droplet formation through alterations in lipid metabolism. For example, *Cyp27a1* controls the generation of the cholesterol derivative 27-hydroxycholesterol, and knockout of the gene is associated with hypertriglyceridemia (38). *Cluster 2* includes the master regulator *Fgf21*, which is highly expressed in neonatal liver and reduced nearly fourfold by CSF1-Fc. Fgf21 has been implicated in control of numerous lipid/obesity-related liver pathologies (25). The bile acid receptors *Nr1h4* (*Fxr*) and Gpbar1 (*Tgr5*), are also within this cluster and might be subject to feedback regulation by elevated bile acids. Many of the genes in *cluster 2*, notably the cytochrome *P*-450 enzymes such as Cyp27a1 and Cyp2e1, have centrilobular enrichment in adult mouse liver (4).

In adult mice, treatment with CSF1-Fc mimicked many effects of insulin, producing a marked downregulation of genes encoding enzymes of gluconeogenesis, fatty acid oxidation, and amino acid catabolism (14). This was not evident in the treated neonatal rats, where CSF1-Fc treatment did not regulate known targets of insulin repression, including the insulin receptor itself (*Insr*), the transcription factor *FoxO1*, and downstream targets such as the signal transducer *Irs2*, gluconeogenic enzymes *Pck1* and *G6pase*, tyrosine amino transferase (*Tat*), *Igfbp1*, and *ApoC2* (58), indicating that insulin signaling is unaltered. We suggest that the difference between the effects of adult and neonatal CSF1-Fc treatments lies in the recruitment of monocytes and inflammatory cytokine production.

In overview, the transcriptional profiling of neonatal rat livers treated with CSF1-Fc highlights many genes that may control lipid droplet formation. Lipid droplet accumulation was not observed in adult mice, or pigs, in response to CSF1-Fc administration (14, 46, 50). In adult animals, CSF1-Fc caused profound monocytosis, and the expanded hepatic macrophage population was predominantly recruited monocytes. The response to CSF1-Fc in the neonates suggests that lipid accumulation is controlled, at least in part, by resident Kupffer cells. In that case, treatment with anti-CSF1R, which rapidly depletes Kupffer cells (26), could have some benefit in reversing lipid accumulation. The focus on lipid droplet formation, which we have shown is rapidly reversible, potentially neglects the potential benefit of such a treatment. In the context of models of acute liver failure, CSF1-Fc treatment rapidly expanded the Kupffer cell population and restored the capacity for removal of potential infections arriving in the portal blood (50). Neonatal sepsis is one of the main causes of morbidity and mortality, attributed in part to the immaturity of the neonatal immune system (48). We have shown that CSF1-Fc treatment of neonatal rats can produce a rapid increase in Kupffer cell numbers independently of monocyte recruitment and regardless of prior maternal stress and LBW. The inducible genes in Supplementary Tables S1 and S2 include numerous pattern recognition and endocytic receptors (e.g., *Siglec1, Siglec5, Nlrp3, Nlrc4, Mrc1, Tlr1,2,4,6,7, Cd163, Clec4a, Msr1, Marco, Timd4, Axl, Vsig4*, and *Fcgr2a*), and the components of the phagocyte oxidase system (*Cyba, Cybb, Ncf2, Ncf1*, and *4*). Accordingly, we suggest that CSF1-Fc treatment has potential to promote rapid maturation of innate immunity in neonates at high risk of infection.

## GRANTS

This work was funded by the Medical Research Council Grant MR/M019969/1. The Roslin Institute also receives Institute Strategic grant funding from the Biotechnology and Biological Sciences Research Council.

## DISCLOSURES

No conflicts of interest, financial or otherwise, are declared by the authors.

## AUTHOR CONTRIBUTIONS

C.P. and D.A.H. conceived and designed research; C.P., K.A.S., G.M.D., L.L., A.R., and R.R. performed experiments; C.P., K.M.I., A.J.N., and D.A.H. analyzed data; C.P., K.M.I., P.B., M.C., and D.A.H. interpreted results of experiments; C.P. and K.M.I. prepared figures; C.P. and D.A.H. drafted manuscript; C.P., K.M.I., A.J.N., and D.A.H. edited and revised manuscript; C.P., K.M.I., and D.A.H. approved final version of manuscript.
